# Supplementary material for: Secretome of Adipose-Derived Stem Cells Cultured in Platelet Lysate Improves Migration and Viability of Keratinocytes
Source: Int J Mol Sci. 2023 Feb 9;24(4):3522. doi: 10.3390/ijms24043522 (PMC9962933; doi:10.3390/ijms24043522)
Supplement: Supplementary file 1 [file ijms-24-03522-s001.zip › ijms-2142371-supplementary.pdf]

Table S1. CD markers in MSC expression.

positive marker	negative marker
CD73	CD11b
CD90	CD19
CD105	CD31
	CD34
	CD45
	HLA-DR

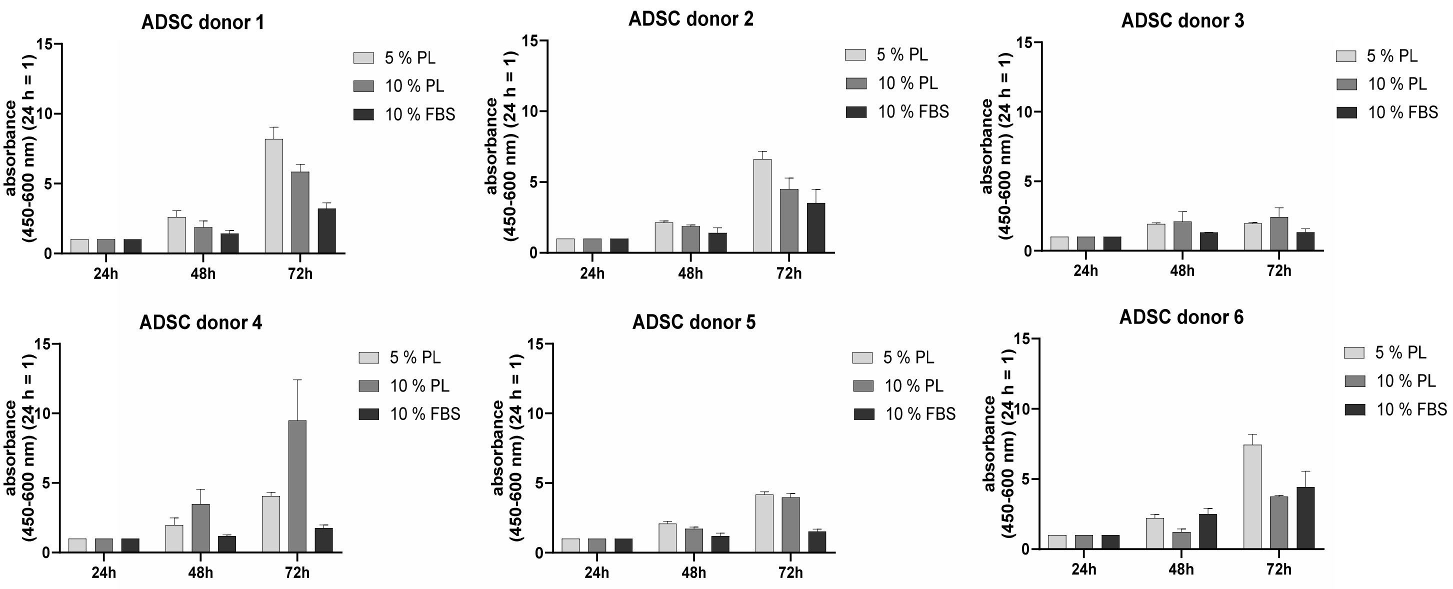

Figure S1. Bar graphs show the relative cell viability of all individual ADSC donors in different groups at 24 - 72 h. The assay was performed in technical triplicates.
